# Short-term results of flanged Bentall de Bono and valvesparing David V procedures for the treatment of aortic root aneurysms

**DOI:** 10.5830/CVJA-2018-021

**Published:** 2018

**Authors:** Ergün Servet, Department of Pediatric Heart Surgery, Dr Siyami Ersek Training and Research Hospital, Istanbul, Turkey, Dedemoğlu Mehmet, Bülent Rabuş Murat, Özbek Baburhan, Mert Özgür Mustafa, Altuğ Tuncer Mehmet, Kaan Kırali Mehmet, Balkanay Mehmet

**Affiliations:** Cardiovascular Surgery Department, Kars Harakani Hospital, Kars, Turkey; Cardiovascular Surgery Department, Kars Harakani Hospital, Kars, Turkey; Cardiovascular Surgery Department, Kars Harakani Hospital, Kars, Turkey; Department of Cardiovascular Surgery, Kartal Kosuyolu Training and Research Hospital, Istanbul, Turkey; Department of Cardiovascular Surgery, Kartal Kosuyolu Training and Research Hospital, Istanbul, Turkey; Department of Cardiovascular Surgery, Kartal Kosuyolu Training and Research Hospital, Istanbul, Turkey; Department of Cardiovascular Surgery, Kartal Kosuyolu Training and Research Hospital, Istanbul, Turkey; Department of Cardiovascular Surgery, Kartal Kosuyolu Training and Research Hospital, Istanbul, Turkey; Department of Cardiovascular Surgery, Izmir Katip Celebi University, Ataturk Training and Research Hospital, Izmir, Turkey

**Keywords:** valve-sparing aortic root surgery, flanged Benthall, David V

## Abstract

**Aim:**

Even though the Bentall de Bono procedure is widely used for the treatment of aortic root aneurysms, the procedure is under scrutiny nowadays because of complications due to mechanical prosthetic valves and the need for life-long anticoagulation. Due to these complications, aortic valvesparing operations are being researched. In this study we compared the short-term morbidity and mortality rates of both Bentall de Bono and valve-sparing David V procedures.

**Methods:**

We retrospectively evaluated data from 70 patients who had undergone surgery for aortic root aneurysm between April 2009 and June 2013. We had performed the Bentall de Bono procedure on 46 patients and the David V procedure on 24 patients. Mortality rates, cardpulmonary bypass (CPB) and aortic cross-clamp durations, postoperative arrhythmias, and prolonged intensive care unit (ICU) and hospital stays were compared in this study.

**Results:**

There was no statistical difference for mortality rate (p = 0.57), while the CPB time and cross-clamp duration were shorter in the Bentall group. When we compared the length of ICU and hospital stay, we observed that the David group stayed longer in ICU (p = 0.003) but the duration of hospital stay was shorter (p = 0.007).

**Conclusion:**

Despite Bentall de Bono being the most commonly used procedure, the short-, mid- and long-term results of both procedures were similar. Spared native aortic valve and lack of anticoagulation usage are notable advantages of the David V procedure.

Several surgical procedures are used to repair ascending aortic aneurysms. Factors such as feasibility, percentage of complications, and surgical morbidity and mortality rates are critically important in choosing the best operation strategy.

Valve-sparing procedures have some advantages, such as not needing to use anticoagulation therapy and there are no complications related to mechanical valves. Procedures using a mechanical composite valved graft also have advantages, such as long-term valve durability.^1^-^5^ There are many discussions about which procedure is better.

The first aortic root replacement with a composite valve graft was performed by Bentall and de Bono in 1968.^6^ In this procedure, the ascending aorta and aortic valve was resected. Firstly a mechanical prosthetic valve was sutured to a tubular graft and then the valved graft was sutured to the aortic annulus with continuous prolene sutures.

A flanged Bentall procedure was reported by Yakut in 2000.^7^ In this procedure about 5 mm of the proximal end of the graft is everted outward. The mechanical valve is fixed to the graft with continuous polyprolene sutures from the stent of the valve to the graft. Then the everted part of the graft is returned to its original position and the conduit is implanted onto the aortic annulus from the flanged part with continuous polyprolene sutures. The rest is the same as with the classical Bentall de Bono procedure.

Classical valve-sparing root replacement was reported by David and Feindel in 1992. They defined their procedure as first resecting the aortic root completely while sparing the valve, preparing the coronary ostia in a button shape, then suturing a dacron graft to the aortic root, and finally suturing the buttons to the dacron graft.^8^

When we assessed the complications related to long-term risk of anticoagulation, we observed the superiority of using homografts, pulmonary otografts, valve-sparing procedures or bioprosthetic valved composite grafts instead of composite grafts with mechanical valves.^4^-^8^

In this study, we evaluated the initial results of the flanged Bentall procedure versus the David V procedure in terms of surgical morbidity and mortality rates, and complications related to the procedures.

We operated on 70 patients (flanged Bentall de Bono: 46, David V: 24) for aortic root aneurysm in our hospital between April 2009 and June 2013. Data were evaluated retrospectively. Patients were divided into two groups according to the procedure. Patients who had the flanged Bentall de Bono procedure were in group 1, and those who had the David V procedure were in group 2.

All patients were intubated under general anaesthesia and they were on mechanical ventilation during the operation. A right jugular central venous catheter was inserted routinely. A standard median sternotomy was performed and cardiopulmonary bypass was instituted. The venous line was inserted through the right atrial appendage generally, but selective bicaval venous cannulation was performed in some cases. Myocardial protection was achieved by cardioplegia, specifically by intermittent antegrade or simultaneous antegrade and retrograde (66.5%) administration of hypothermic blood cardioplegia, according to the decision of the surgeon. A venting cannula was inserted through the right superior pulmonary vein.

In group 1 we performed a modified version of the Bentall procedure, called a flanged Bentall. In this procedure, the ascending aorta is resected and the coronary ostia are prepared as buttons. Then the aortic root and valve is resected. A mechanical aortic valve is sutured to the composite graft, leaving a part of the proximal side everted outwards (flange). This is done with continuous polyprolene sutures from the stent of the valve to the graft. The conduit is sutured to the aortic annulus with polyprolene sutures from the flanged part. After this, the coronary buttons are sutured to the graft. The distal part of the graft is then sutured to the distal aorta with teflon felt.

In group 2, a routine transverse aortotomy was done about 2 cm above the coronary ostia. If it was clear that there was no structural pathology on the aortic valve, the ascending aorta was resected, keeping 5 mm of aortic tissue on the aortic annulus. Thereafter the standard David V procedure was performed.

## Statistical analysis

Statistical analyses were performed using SPSS version 15.0 software. Compliance with the normal distribution of variables was evaluated with visual (histogram and probability graphics) and analytical methods (Kolmogorov–Simirnov/Shapiro–Wilk tests). Descriptive analysis was done using frequency tables for categorical variables and means and standard deviations for normally distributed variables. We used the median and 25th and 75th quartiles for analysis of variables without a normal distribution. To compare the groups, we performed an independent samples t-test for variables with normal distribution, and the Mann–Whitney U-test for those without a normal distribution. We also performed Pearson’s chi-squared test (χ2) for categorical variables; p < 0.05 was considered as significant.

## Results

The mean age of the patients was 52.01 ± 16.82 years, and 52 (74.3%) were male and 18 (25.7%) were female. There were statistically significant differences between the groups in terms of age and having coronary artery disease (p = 0.04 and p < 0.001, respectively). Demographic features of the groups are summarised in Table 1.

**Table 1:** Demographic data of the two groups

*Demographics*	*Group 1 (n = 46)*	*Group 2 (n = 24)*	*p-value*
Age	50.04 ± 18.05	58.70 ± 12.67	0.04
Male gender, n (%)	35 (76)	17 (70.8)	0.63
COPD, n (%)	16 (34.7)	10 (41.6)	0.57
Smoking, n (%)	14 (30.4)	12 (50)	0.10
Hypertension, n (%)	26 (56.5)	18 (75)	0.12
Diabetes mellitus, n (%)	15 (32.6)	6 (25)	0.51
Coronary artery disease, n (%)	2 (4.3)	9 (37.5)	< 0.001
Peripheral vascular disease, n (%)	2 (4.3)	0 (0)	0.54
NYHA class 1, n (%)	8 (17.3)	4 (16.6)	
NYHA class 2, n (%)	12 (26.08)	7 (29.1)	< 0.06
NYHA class 3, n (%)	14 (30.4)	7 (29.1)	
NYHA class 4, n (%)	12 (26.08)	6 (25)	

COPD: chronic obstructive pulmonary diesase, NYHA: New York Heart Association.

When we compared the pre-operative echocardiographic findings, there was a statistically significant difference in the aortic annulus diameter between the groups (p = 0.03). Mean aortic annulus diameter was 3.30 ± 1.32 cm in group 1 and 2.76 ± 0.41 cm in group 2. Mean ascending aorta diameter was 5.49 ± 1.18 cm in group 1 and 5.73 ± 0.99 cm in group 2 (p = 0.39). There was no significant difference in terms of degree of aortic insufficiency between the groups (p > 0.05) (Table 2).

**Table 2:** Pre-operative echocardiographic findings

*Pre-operative echo findings*	*Group 1 (n = 46)*	*Group 2(n = 24)*	*p-value*
Ascending aorta diameter	5.49 ± 1.18	5.73 ± 0.99	0.39
Aortic annulus diameter	3.30 ± 1.32	2.76 ± 0.41	0.03
Sinus of Valsalva diameter	5.16 ± 1.17	5.08 ± 0.69	0.79
EF (%)	60.85 ± 7.4	57.50 ± 10.57	0.42
LVESD (cm)	3.74 ± 1.04	5.45 ± 7.8	0.17
LVEDD (cm)	5.43 ± 1.06	7.98 ± 11.59	0.16
IVS (cm)	1.25 ± 0.19	1.11 ± 0.12	0.50
AI, n (%)	31 (67.3)	19 (79.1)	0.30
AI degree			0.13
1, n (%)	7 (15.2)	0 (0)	
2, n (%)	11 (23.9)	10 (41.6)	
3, n (%)	4 (8.6)	4 (16.6)	
4	9 (19.5)	5 (20.8)	

Echo: echocardiography, EF: ejection fraction, LVESD: left ventricular endsystolic diameter, LVEDD: left ventricular end-diastolic diameter, IVS: interventricular septum, AI: aortic insufficiency, cm: centimetre.

When we compared surgical data (Table 3), average cardiopulmonary bypass time was 69.55 min shorter in group 1 (p = 0.001, 95% CI: 108.19–30.90 min). Average arterial crossclamp time was 80.75 min shorter in group 1 (p < 0.001, CI: 101.55–59.95 min).

**Table 3:** Surgical data between groups

*Surgical data*	*Group 1 (n = 46)*	*Group 2 (n = 24)*	*p-value*
TPT (min)	134.84 ± 86.93	204.39 ± 44.7	0.001
ACC (min)	83.11 ± 41.64	163.86 ± 38.06	< 0.001
TCA (min), n (%)	0 (0)	4 (16)	0.004
Hypothermia (°C)	28.21 ± 1.57	28.1 ± 2.73	0.86
Cannulation side			< 0.001
Axillary, n (%)	44 (95.6)	6 (25)	
Aortic, n (%)	1 (2.2)	17 (70.8)	
Brachiocephalic, n (%)	0 (0)	1 (4.2)	
Femoral, n (%)	1 (2.2)	0 (0)	

TPT: total perfusion time, ACC: arterial cross-clamping time, TCA: total circulatory arrest.

With regard to postoperative complications, postoperative arrhythmias were seen in four (8.6 %) and one (4.2%) patient in groups 1 and 2, respectively (p = 0.004). There was no statistically significant difference between the groups in terms of respiratory complications, bleeding requiring re-exploration, and intraaortic balloon pump (IABP) usage. No stroke was observed in either group.

We lost one patient in each group and there was no statistically significant difference between the groups in terms of mortality rate. ICU stay was shorter in group 1. Average ICU stay was 1.85 ± 1.02 days in group 1 and 3.70 ± 3.07 days in group 2 (p = 0.003). Duration of hospital stay was longer in group 1 (average 14.64 ± 3.52 days, p = 0.007) (Table 4).

**Table 4:** Postoperative complications and length of hospital stay of the two groups

*Postoperative complications*	*Group 1 (n = 46)*	*Group 2 (n = 24)*	*p-value*
Bleeding revision, n (%)	0 (0)	1 (4.2)	0.34
Respiratory, n (%)	2 (4.3)	1 (4.2)	0.73
IABP usage, n (%)	1 (2.2)	0 (0)	0.65
Arrhythmias, n (%)	4 (8.6)	1 (4.2)	0.004
Mortality, n (%)	1 (2.2)	1 (4.2)	0.57
ICU stay (days)	1.85 ± 1.02	3.70 ± 3.07	0.003
Hospital stay (days)	14.64 ± 3.52	12.20 ± 8.08	0.007

IABP: intra-aortic balloon pump, ICU: intensive care unit.

On postoperative echocardiography, we observed that degree of aortic insufficiency was significantly decreased in patients who had undergone the David V procedure (p < 0.001). No moderate or severe aortic insufficiency was detected (Table 5).

**Table 5:** Pre-operative and postoperative parameters related to aortic insufficiency in the David V group

*Pre-operative AI (median/min–max)*	*Postoperative AI (median/min–max)*	*p-value*
2.5 ( 2–4)	0 (0–2)	< 0.001

AI: aortic insufficiency.

When we compared factors related to mortality rate, age, hemi-arch replacement as an additional procedure, ascending aortic diameter and hypothermia were important parameters. We observed that advanced age (p = 0.04), wide ascending aortic diameter (p = 0.04), hypothermia (p = 0.001) and hemiarch intervention (p = 0.01) increased mortality rates (Table 6). Age (p = 0.01), ascending aortic diameter (p = 0.02), degree of hypothermia (p < 0.001) and hemi-arch replacement (p = 0.001) were factors that negatively affected ICU stay (Table 6).

**Table 6:** Parameters related to mortality and ICU stay

*Parameters*	*Correlation coefficient*	*p-value*
Parameters related to mortaliy		
Age	0.23	0.04
Diameter of ascending aorta	0.35	0.004
Hypothermia	(–) 0.39	0.001
Hemi-arch replacement	0.28	0.01
Parameters related to ICU stay
Age	0.31	0.01
Diameter of ascending aorta	0.29	0.02
Hypothermia	(–) 0.54	< 0.001
Hemi-arch replacement	0.42	0.001

ICU: intensive care unit.

We performed mitral reconstruction in four (5.7%) patients, mitral valve replacement in three (4.3%), atrial septal defect repair in two (2.9%), radiofrequency ablation in one (1.4%), hemi-arch replacement in five (7.1%) and coronary artery bypass grafting (CABG) in eight (11.4%) patients as an additional procedure, out of a total of 70 patients. In addition to these findings, aortic cross-clamp and cardiopulmonary bypass time were significantly longer in patients who underwent CABG as an additional procedure (p < 0.001, p = 0.007, respectively).

## Discussion

The Bentall de Bono procedure is seen as the gold-standard surgical choice for the treatment of standard aortic root aneurysms but because of complications (mortality, morbidity) related to the use of anticoagulants when mechanical valves are used, valve-sparing procedures have come into prominence. When we look at initial and mid-term results of valve-sparing procedures, there is no doubt that these techniques can be confidently used for aortic root aneurysms.^1^,^2^,^4^

An important study on this subject was done by Gaudino et al.^4^ in 890 patients, where 289 mechanical valve composite grafts, 421 biological valve composite grafts and 180 valve-sparing procedures were evaluated. The surgical mortality rate was 0.2% (none for the valve-sparing procedures).

They observed that length of hospital stay was related to age, emergency operation, renal function, re-operation, New York Heart Association class, ejection fraction, and additional procedures. At five years, follow-up survival was 89.4%. Renal function, previous myocardial infarction, redo operations and additional procedures were related to long-term survival. They observed no significant difference regarding early and longterm mortality rates between the groups. At five years, the need for follow-up re-operation was 0% for the mechanical valve composite grafts, 2.4% for biological valve composite grafts and 7.3% for valve-sparing procedures.^4^ We noticed that valve durability was of importance in the mechanical valve graft group in that study.

In our study, older age (p = 0.04), wide ascending aortic diameter (p = 0.004), less degree of hypothermia (p = 0.001) and additional hemi-arch replacement (p = 0.001) were the parameters that increased early period mortality rates. Additionally, advanced age (p = 0.01), wide ascending aortic diameter (p = 0.02), degree of hypothermia (p < 0.001) and additional hemiarch replacement (p = 0.001) also increased length of ICU stay.

In a recent study, Lamana et al.^9^ analysed 324 patients, of whom 263 had undergone mechanical composite valve graft surgery and 61 valve-sparing root surgery. They observed that there were no statistically significant differences in short-term mortality rates (p = 0.71), but the long-term mortality rate was lower in the valve-sparing group (p = 0.001). In the same study, they noticed that bleeding (requiring re-exploration) was lower in the valve-sparing group and they associated the higher mortality rate in the composite valve graft group with early and late-term complications related to bleeding. Additionally, there were significant differences between the groups in terms of thromboembolic events in that study.^9^

Coselli et al.^10^ studied early and mid-term results of 83 patients (82 re-implantations, one Florida sleeve procedure). They observed one surgical mortality, one stroke (because of acute aortic dissection) and intra-operative valve replacement was performed on one patient. They concluded that valvesparing procedures had satisfying early period results.^10^

Arabkhani et al.^11^ studied 4 777 patients and 1 659 articles in a meta-analysis. They compared valve-sparing procedures (72% re-implantation, 27% remodelling, 1% other procedures) and indicated that early period mortality rate was 2%, and there were no statistically significant differences between the procedures in terms of survival and re-operation. They concluded that valvesparing procedures were an alternative to mechanical valve grafts in terms of survival rate.^11^

Patel et al.^12^ studied 140 patients (56 Bentall de Bono, 84 valve-sparing procedures). They implied that thromboembolic events were higher in the Bentall group. Additonally eight-year survival rate was higher in the valve-sparing group.^12^

David et al.^5^ studied 296 patients who underwent re-implantation procedures. They observed four surgical deaths and 18 late-term deaths. Five-year survival rate was 95.1 ± 3.5%, 10-year survival was 93.1 ± 4.4% and 15-year survival was 76.5 ± 18%. They performed mechanical composite valve grafts on three patients because of severe aortic insufficiency.^5^

Kallenbach et al.^13^ and Svensson et al.^14^ showed similar results in their studies. Parallel to these findings, in our study, there was no statistically significant difference between the Bentall de Bono and David V procedures in terms of morbidity and mortality rates (p = 0.57).

Karendi et al.^15^ compared the David procedure (37 patients) with the Bentall de Bono procedure (73 patients) in highrisk patients (type A aortic dissections, re-operations). They indicated that there was no statistically significant difference between the procedures related to pre-operative and operative data, except for cross-clamp time.^15^ Similar to this, in our study, CPB time and cross-clamp time were significantly longer in the David V group but there was no statistically significant difference in other surgical data between the groups.

In our study there was no statistically significant difference between the groups in terms of in-hospital mortality rate, bleeding (requiring re-exploration), thromboembolic events, respiratory complications and IABP usage. Incidence of cardiac arrhythmias was higher in the Bentall group (p = 0.004). There were no operative deaths in either group. We lost one patient in each group because of pneumonia in the early period.

Skripochnik et al.^16^ studied 70 patients (25 valve-sparing, 45 Bentall procedure) and observed no difference between the groups regarding peri-operative mortality rates. In addition, there was no significant difference between the groups in terms of length of hospital and ICU stay.^16^

In our study, ICU stay was longer (p =0.003), but hospital stay was shorter in the David V group (p = 0.007). We believe that long hospital stay for the Bentall group was associated with warfarin use and dose adjustments, because we routinely follow patients at in-patient clinics until appropriate INR values are achieved. In addition, we found that age (p = 0.01), diameter of ascending aorta (p = 0.002), hypothermia (p < 0.001) and hemiarch replacement (p = 0.001) had an impact on length of ICU stay. We believe that longer ICU stay for the David V group was associated with longer cross-clamp time, longer total perfusion time (p = 0.001) and higher average age (p = 0.04).

## Conclusion

Although the Bentall de Bono procedure is seen as the goldstandard surgical approach for aortic root aneurysms, there are some complications related to this procedure. Because the native aortic valve is not protected in the dilated aortic root, physiological superiority of the native valve is lost. In addition, warfarin use and dose adjustment prolongs hospitay stay.

Many studies have revealed that early, mid- and long-term results of the Bentall and David V procedures are similar. Since there was no difference between the two procedures in our study in terms of morbidity and mortality rates, the biggest advantage of the David V procedure is that it protects the native valve. In addition, the David V procedure is safer because there are no complications related to the use of mechanical valves and anticoagulants in the postoperative period. Therefore we propose use of the David V procedure primarily for patients with dilated aortic root and/or dilated ascending aorta with normal aortic valves.
